# Tattoo Guidelines in the At-Risk Cancer Population

**DOI:** 10.7759/cureus.37495

**Published:** 2023-04-12

**Authors:** Dawson Foster, Joseph Sokhn

**Affiliations:** 1 Internal Medicine, St. Luke's Hospital, Chesterfield, USA; 2 Hematology and Oncology, St. Luke's Hospital, Chesterfield, USA

**Keywords:** high-risk group, cancer patients, cancer surveillance, cancer, tattoo pigment, prevention of cancer

## Abstract

With tattoo prevalence on the rise in all age groups, it is important to acknowledge that it is a potential cause of lymphadenopathy while simultaneously being aware of its mimicking presence in high-risk populations such as those with current or prior cancer diagnoses. The period of time between identification and diagnosis provides a great amount of stress and anxiety for patients and their families. We present a case of a patient who had multiple recurrences of an unknown primary and underwent multiple workups with no subsequent diagnosis. One particular workup yielded the diagnosis of tattoo-related lymphadenitis; while this particular occurrence was a benign finding, the extensive workup took a toll on the patient and his family as the fear of cancer progression with an allusive diagnosis continued to be a major factor in their lives.

## Introduction

In the 21st century, the stigma against body art has lessened and as a result, there has been an increase in the number of people who seek to acquire tattoos. As the population of people that have permanent body art increases the presence of adverse events and future unseen outcomes increase, tattoo ink has been noted in nearby lymphatic clusters and has even been known to spread to distant clusters. Cases of tattoo-related lymphadenitis have become well-documented. While benign, this process can cause significant stress in specific populations as the threat of a cancer diagnosis looms with lymphadenopathy. To populations such as those who have or have had cancer the ultimate outcome can be life-altering. In addition, patients with risk factors for the development of cancer be they genetics or lifestyle will require a workup for acute lymphadenopathy. During this workup, patients will be between a diagnosis of cancer free and a diagnosis that will change their life forever. This purgatory will provide significant stress for patients and their families while the uncertain etiology of lymphadenopathy is pending. We present a case of a patient with dormant cancer who despite multiple recurrences and treatments no specific diagnosis was discovered. This particular workup yielded the diagnosis of tattoo-related lymphadenitis due to a recently acquired tattoo; while this particular occurrence was a benign finding the extensive workup took a toll on the patient and his family as the fear of cancer progression with an allusive diagnosis continued to be a major factor in their lives. 

## Case presentation

A 66-year-old male presented to the emergency room with new onset crushing lower back pain and mild right upper quadrant pain. Workup identified concerning liver lesions, porta-hepatic lymphadenopathy, and lytic lesions of L3 and L4 lumbar vertebrae. The patient was diagnosed with stage 4, undifferentiated carcinoma of unknown primary. He was subsequently treated with stereotactic radiation. Multiple biopsies were attempted at varying sites yielding the same inconclusive results due to either poor differentiation or issues of sampling error. After completing stereotactic radiation at the affected sites, follow-up computed tomography (CT) scan images showed a decrease in lymph node and lesion sizes.

While undergoing subsequent surveillance, a CT scan identified a new para-esophageal lymph node. A biopsy was performed and was again diagnosed as poorly differentiated carcinoma, with no further classification possible. A positron emission tomography (PET) CT scan was performed and avid fluorodeoxyglucose (FDG) PET uptake was noted in the para-esophageal node as well as a posterior mediastinal node. The patient was treated with stereotactic radiation to both sites and the lesions responded well to the treatment.

**Figure 1:**
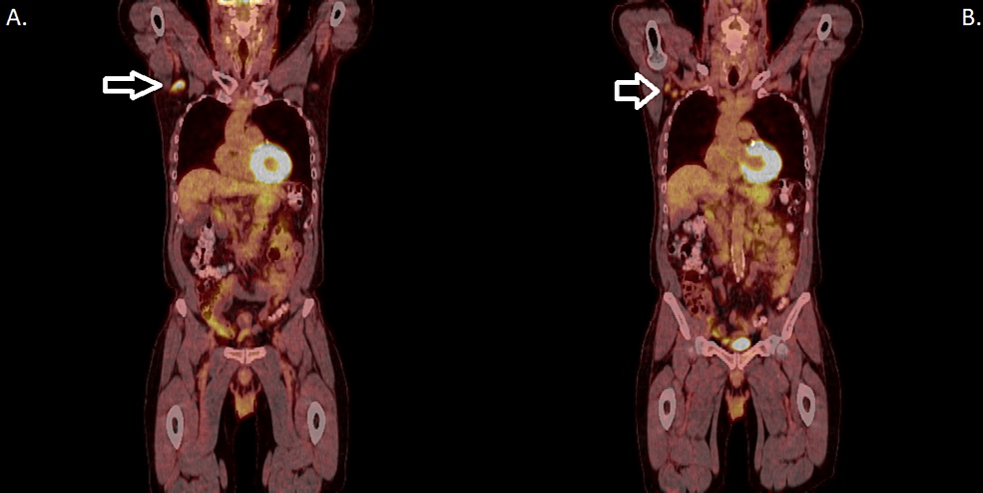
PET CT scan with FDG uptake A highlights the greatest area of uptake while B highlights the three nodes of interest. PET: positron emission tomography, FDG: fluorodeoxyglucose

Eight months later, the patient noted swelling in both left and right axillary lymph nodes, the right greater than the left. PET CT scan was again performed and avid FDG uptake was noted in the right axilla. A large node was noted in Figure 1A with three total nodes present in Figure 1B. An excisional biopsy was performed. The patient and family emotionally struggled with the possibility of cancer recurrence.

**Figure 2:**
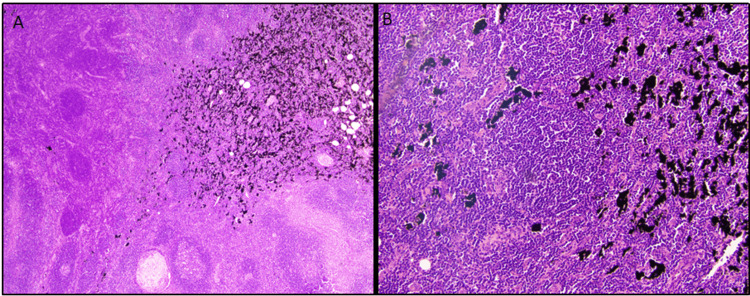
Ink noted within slide preparation A highlights the tattoo-related lymphadenitis with deposits of tattoo ink at a 40x magnification while B shows the disturbed architecture with tattoo ink at 200x magnification.

The pathologic sample revealed acute lymphadenitis with reactive follicular hyperplasia. There was an expanded inter-follicular area mixed with histiocytes, neutrophils, apoptosis debris, and abundant ink pigment seen in Figure 2. All features were indicative of acute lymphadenitis secondary to tattoo ink, with no signs of malignancy found. 

During the follow-up visit, a recently acquired tattoo was identified on the patient's back. It extended from one arm traversing the upper and middle back to the opposite arm. The family was relieved but frustrated by the lack of clear cancer origin.

## Discussion

The regulation of tattoo ink by the federal government is still loosely defined and rarely enforced. Manufacturers at times fail to disclose their contents [1,2]. The components of the ink can range from inorganic metallic salts, varying organic molecules, and dyes some of which may cause a hypersensitivity reaction while others may even be carcinogenic [3-7]. Due to the lack of enforced oversight, varying carrier molecules, solvents, additives, and metals are used for pigmentation. It is difficult to predict who may develop lymphadenopathy. While these cases are rare, this example is not unique in highlighting the possible risk of acquiring a tattoo [8-10].

It is also difficult to predict the timeline of events. Components like cadmium, chromium, cobalt, mercury, and nickel in the tattoo have been well-documented as potential causes of delayed reactions [7,11,12]. As with our case, not only does the physical exam finding of lymphadenopathy mimic cancer progression, but tattoo-related lymphadenitis can also lead to a false positive read on the PET/CT scan and another biopsy [13]. This process will cause further stress and anxiety for the patient, family, and medical team. While the cause of the lymphadenopathy is ultimately benign it can take weeks from the initial identification to the concluding pathology report. We recommend patients with a high risk for cancer and those who have been or are currently diagnosed with cancer should follow the guidelines proposed in Table 1. 

**Table 1:** Recommended Tattoo-Acquiring Guidelines Proposed guidelines prior to acquiring the tattoo will help to limit adverse outcomes.

Shared Decision Topics and Work Up	Indicated Reason
Tattoo Acquisition should be a shared decision process	Allow the patient to his or her bodily autonomy while providing adequate insight into the possible adverse outcomes
Discuss the intended location	Physician aware of possible involved lymphatic clusters
Check complete blood count (CBC) and coagulation times before the procedure	Help assess bleeding risk and signs of possible infection
Have tattoo done after a recent surveillance CT scan	This can provide a baseline if new lymphadenopathy develops after the procedure
Acquire the specifications of the tattoo artist’s ink	This may identify any components that the patient may react too

## Conclusions

As the percentage of the population with tattoos grows so will tattoo-related lymphadenitis. While the general population is aware of adverse events like local irritation and the rare risk of infections they are likely not aware of tattoo-related lymphadenitis. It is important to educate people that are at high risk for cancer and those with current and/or prior diagnoses that acquiring a tattoo can not only increase one’s risk for infection and bleeding but also be a mimicker of cancer and cancer progression. In this case, the tattoo-related lymphadenitis was benign but the workup and concern for cancer progression were not.
